# A Scoping Review of the Mechanisms Influencing Socioeconomic Disparities in Outcomes of Digital Interventions for Weight‐Related Behaviors

**DOI:** 10.1111/obr.70121

**Published:** 2026-03-12

**Authors:** Lee C. Mercer, Mirna Al‐Masri, Diana Rocha, Laura M. König, Max Western

**Affiliations:** ^1^ Department of Health Centre for Motivation and Health Behavior Change University of Bath Bath UK; ^2^ Faculty of Life Sciences: Food, Nutrition and Health, Department of Health University of Bath Bath UK; ^3^ Faculty of Law, Business and Economics University of Bayreuth Bayreuth Germany; ^4^ Faculty of Psychology University of Vienna Vienna Austria

**Keywords:** digital divide, digital health, health inequalities, socioeconomic status

## Abstract

Digital behavioral change interventions (DBCIs) for weight‐related behaviors may be less effective in disadvantaged populations, potentially widening health inequalities despite increased access. Limited research has explored the psychosocial mechanisms that may contribute to this divide. Following JBI guidelines for conducting scoping reviews, we conducted an electronic search on Embase, PubMed, APA PsycInfo, Web of Science, and SCOPUS on March 8, 2023, including studies published since 1990. The mechanisms of action ontology was used for deductive coding of the mechanisms discussed. The protocol was registered on the Open Science Framework (https://osf.io/ctua5). After initial screening of 17,503 papers, 21 studies met inclusion criteria, including RCTs, pre‐post studies, systematic reviews, qualitative studies, cross‐sectional, pilot, and feasibility studies. A second screen of 7840 articles in June 2025 identified three further studies that met the inclusion criteria. Socioeconomic inequalities and ethnicity were the predominant focus. Environment, motivation, and social influences were frequently cited mechanisms. However, mechanisms are inconsistently conceptualized and measured, highlighting a gap in explanatory research on the digital health divide.

## 1 Introduction

Health inequality remains a persistent and pressing challenge in public health and is a priority in the World Health Organization's General Programme of Work for 2019–2024 [1]. For example, socioeconomic status (SES)—a composite measure consisting of income, education, and occupation—has long been understood to be positively correlated with health outcomes across multiple domains of health [2, 3, 4, 5, 6, 7, 8, 9]. One prominent health outcome influenced by socioeconomic factors is obesity [5] making it a focus of public health interventions aimed at changing health behaviors. Typically, behavioral change interventions (BCIs) are a set of structured activities, which seek to modify behaviors by targeting psychosocial factors such as motivation, intention, self‐efficacy, and social norms [10], and there is a consistent evidence base suggesting that BCIs can be effective at promoting weight loss [11, 12, 13, 14]. Individual and intervention‐related factors, as well as psychological processes, such as personal agency, appear to be important predictors of weight loss from BCIs [13, 15, 16]. These factors likely favor groups with greater access to personal resources including material, temporal, mental, social, and cultural, and there is limited evidence that BCIs may be differentially effective across indicators of inequalities, including SES [17, 18]. This suggests that dedicated strategies may be necessary for BCIs targeting disadvantaged groups so as not to exacerbate existing health inequalities [18].

Disparities also exist in the digital domain [19, 20]. While research on the digital divide has largely focused on the first and second level digital divides (access and skills respectively) [21, 22], less attention has been paid to disparities of outcomes, often described as the third level digital divide [23, 24]. Digital behavioral change interventions (DBCIs) for Health are BCIs delivered through technologies such as the Internet, smartphones, and smartwatches, which target health behaviors. DBCIs have been employed to tackle weight‐related behaviors including physical activity (PA), sedentary behavior, and diet [25, 26]. While evidence suggests that DBCIs targeting weight‐related behaviors may be effective in the short term, their long‐term efficacy remains uncertain [27, 28, 29, 30]. Notably, tailored digital interventions appear more effective than “one‐size‐fits‐all” approaches [31, 32, 33]. Inequalities can arise in access to digital health technologies [34, 35], and digital skills are unevenly distributed, contributed to by socioeconomic factors such as occupation and education level [22, 36, 37]. However, the evidence base on differences in outcomes is mixed. While some studies suggest that digital health can be effective at promoting weight loss in disadvantaged populations [18, 38], others suggest clear outcome disparities [39]. However, the evidence base is limited by inconsistent reporting of outcomes by inequality indicators despite the existence of frameworks such as PROGRESS‐Plus (place of residence, race/ethnicity, occupation, gender, religion, education, SES, social capital, plus other discriminating factors) [18, 39, 40, 41]. Despite this, proponents of digital health argue that the accessibility and reach of digital technology and the Internet make digital tools well placed to tackle existing health inequalities [42].

Researchers have drawn on several theories to help explain the digital divide, including technology acceptance models [43, 44], diffusion of innovation [45], and theories from cognitive and behavioral science such as Social Cognitive Theory [46] and the Theory of Planned Behavior [46, 47]. An understanding of the digital health divide likely requires insights into both how different groups adopt technology and the socio‐cognitive constructs such as self‐efficacy, intention, planning, and outcome expectancy that drive behavior change [48, 49]. Recently, ecological approaches have helped frame how environmental and social contexts give rise to digital health inequalities [50, 51]. For example, Jahnel and colleagues' *Digital Health Rainbow* (2023) [52] explores the hierarchy of digital determinates of health from individual factors, to social and community networks, and the general socioeconomic, cultural and environmental contexts. However, more evidence is needed about the causal mechanisms that underpin the relationship between determinant and outcome. A causal mechanism can be broadly understood as a step or process that causes a specific change [53]. In behavioral science, the term “mechanisms of action” has been adopted to describe the processes through which a behavior change technique affects a real world behavior [54]. Examples include self‐monitoring, goal setting, personalized feedback, participant engagement, psychological empowerment, persuasion, digital literacy, self‐efficacy, and trust [55]. However, there is little standardization in how mechanisms are conceptualized [56]. A better understanding of the mechanisms that help deliver DBCI outcomes, particularly in disadvantaged populations, would be beneficial.

This scoping review is exploratory and descriptive. It aims to examine how researchers have discussed and sought to explain DBCI outcome disparities through the mechanisms that underpin them. The review will map the available evidence, identify gaps, and propose directions for future research. Evidence will be synthesized using narrative analysis, with additional post hoc coding of mechanisms described using the mechanisms of action (MoA) ontology, a classification system to define and categorize MoAs and their relationships [57]. To the best of our knowledge, the MoA ontology is the only peer‐reviewed classification system for categorizing MoA in BCIs (Table 1).

**TABLE 1:** Mechanisms of action (Schenk et al. 2024 [58]).

MoA group	Definition
Knowledge	An awareness of the existence of something.
Skill	An ability or proficiency acquired through practice.
Social/professional role and identity	A coherent set of behaviors and displayed personal qualities of an individual in a social or work setting.
Beliefs about capabilities	Beliefs about one's ability to successfully carry out a behavior.
Optimism	Confidence that things will happen for the best or that desired goals will be attained.
Beliefs about consequences	Beliefs about the consequences of a behavior (i.e., perceptions about what will be achieved and/or lost by undertaking a behavior, as well as the probability that a behavior will lead to a specific outcome).
Reinforcement	Processes by which the frequency or probability of a response is increased through a dependent relationship or contingency with a stimulus or circumstance.
Intention	A conscious decision to perform a behavior or a resolve to act in a certain way.
Goals	Mental representations of outcomes or end states that an individual wants to achieve.
Memory, attention, and decision processes	Ability to retain information, focus on aspects of the environment, and choose between two and more alternatives.
Environmental context and resources	Aspects of a person's situation or environment that discourage or encourage the behavior.
Social influences	Those interpersonal processes that can cause oneself to change one's thoughts, feelings or behaviors.
Emotion	A complex reaction pattern involving experiential, behavioral, and physiological elements.
Behavioral regulation	Behavioral, cognitive, and/or emotional skills for managing or changing behavior.
Norms	The attitudes held and behaviors exhibited by other people within a social group.
Subjective norms	One's perceptions of what most other people within a social group believe and do.
Attitude toward the behavior	The general evaluations of the behavior on a scale ranging from negative to positive.
Motivation	Processes relating to the impetus that gives purpose or direction to behavior and operates at a conscious or unconscious level.
Self‐image	One's conception and evaluation of oneself, including psychological and physical characteristics, qualities, and skills.
Needs	Deficit of something required for survival, well‐being, or personal fulfillment.
Values	Moral, social, or aesthetic principles accepted by an individual or society as a guide to what is good, desirable, or important.
Feedback processes	Processes through which current behavior is compared against a particular standard.
Social learning/imitation	A process by which thoughts, feelings, and motivational states observed in others are internalized and replicated without the need for conscious awareness.
Behavioral cueing	Processes by which behavior is triggered from either the external environment, the performance of another behavior, or from ideas appearing in consciousness.
General attitudes/beliefs	Evaluations of an object, person, group, issue or concept on a scale ranging from negative to positive.
Perceived susceptibility/vulnerability	Perceptions of the likelihood that one is vulnerable to a threat.

The review will seek to answer the following research question: What MoAs have been reported to explain socioeconomic differences in the engagement and efficacy of digital behavior change interventions for weight‐related behaviors?

## 2 Materials and Methods

### 2.1 Design

The review followed the Joanna Briggs Institute (JBI) methodology for conducting scoping reviews [59]. In addition, this report complies with Preferred Reporting Items of Scoping Reviews (PRISMA‐ScR) [60]. The study protocol was uploaded to the Open Science Framework (OSF) on January 21, 2023 (https://osf.io/kgyfw/).

### 2.2 Eligibility Criteria

Eligibility criteria were developed using the Population, Concept, and Context (PCC) Framework [58] (Table 2).

**TABLE 2:** Eligibility criteria.

**Population**	Studies conducted with adults aged 18 or over. Studies that either focus on a socioeconomically disadvantaged population, or compare advantaged and disadvantaged populations in a subgroup analysis. Given anticipated differences in context in countries for various strata we have elected not to formally define thresholds or cutoff points for defining higher or lower SES.
**Concept**	Studies that explore differences in the effectiveness of DBCIs targeting weight‐related behaviors (e.g., diet and physical activity) related to SES or its correlates. DBCIs must primarily be delivered via the Internet, smart phones, tablets, and wearables and intended for use by individuals not healthcare professionals. Interventions delivered primarily by text message were excluded. Hybrid studies, which included nondigital components, are included provided the majority of the intervention content was delivered via digital technology.
**Context**	Studies before 1990, i.e., prior to the widespread use of the Internet, were excluded. Only studies published in the English language were included. There were no restrictions based on the country of origin of the research. Scoping reviews and other gray literature including conference abstracts, study protocols, and editorials were excluded.

### 2.3 Search Strategy and Selection Criteria

The authors created a search strategy with a subject librarian to identify articles, which included reference to the following four core concepts: health, digital, inequalities, and efficacy. The search adopted MESH terms, keywords, and synonyms. Search strategies were adapted to reflect specific terminologies and search architecture of the database (see supplementary materials). An electronic search was conducted of the following databases: Embase (embase.com interface), PubMed (including MEDLINE), APA PsycInfo (APA PsycNet interface), Web of Science (Core Collection), and SCOPUS. Publications were uploaded to Covidence (covidence.org), an online software program to help manage the screening process. Duplicates were identified and removed automatically by Covidence. The lead author (L.C.M.) conducted the initial electronic search on March 8, 2023. L.C.M. also conducted additional backward citation tracing on the three systematic review papers, which met the inclusion criteria in April 2024. An update to the search was conducted in June 2025.

### 2.4 Selection and Screening Process

In the first round, all articles were screened by two of the three authors (L.C.M., M.A.‐M., or V.R. for the initial search; L.C.M., L.M.K., or M.W. for the updated search) against the eligibility criteria set out in the scoping review protocol. Where disagreements were not resolved by the authors, L.M.K. and M.W. provided further guidance or arbitration. Following the initial screen, L.C.M. and M.A.‐M. conducted full‐text reviews on the initial retrieved studies, and L.C.M. and M.W. reviewed full texts for the updated search. The reasons for exclusion from the final review were recorded in Covidence.

### 2.5 Data Extraction and Summary

Data related to the population, interventions, outcomes, and mechanisms identified were extracted by the lead author into a Microsoft Excel spreadsheet (Version 2405). A second reviewer (M.A.‐M.) performed quality checking of the extracted data. Article selection, screening, and data extraction were completed by February 8, 2024. A second search was conducted on June 18, 2025. Extracted data were summarized and tabulated. In addition, L.C.M. and M.W. conducted post hoc deductive categorization of the mechanisms that were either discussed or formally measured against the MoA Ontology [57], a descriptive framework setting out the process through which interventions change behaviors. Coding was conducted using nVivo (Version 1.7 [1533]).

## 3 Results

**FIGURE 1:**
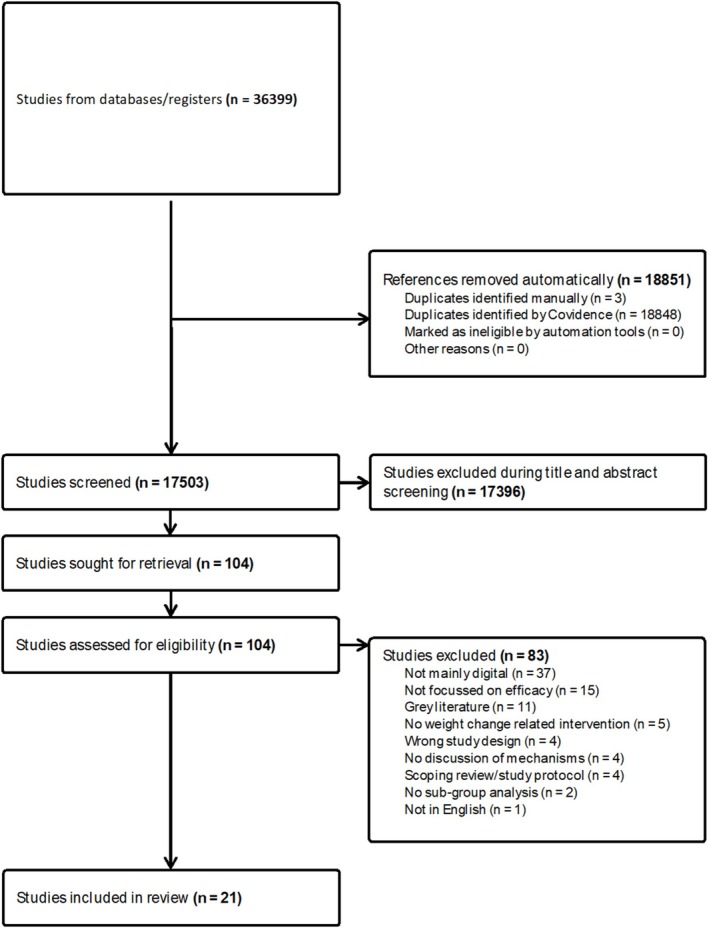
PRISMA‐ScR diagram (search conducted in March 2023).

**FIGURE 2:**
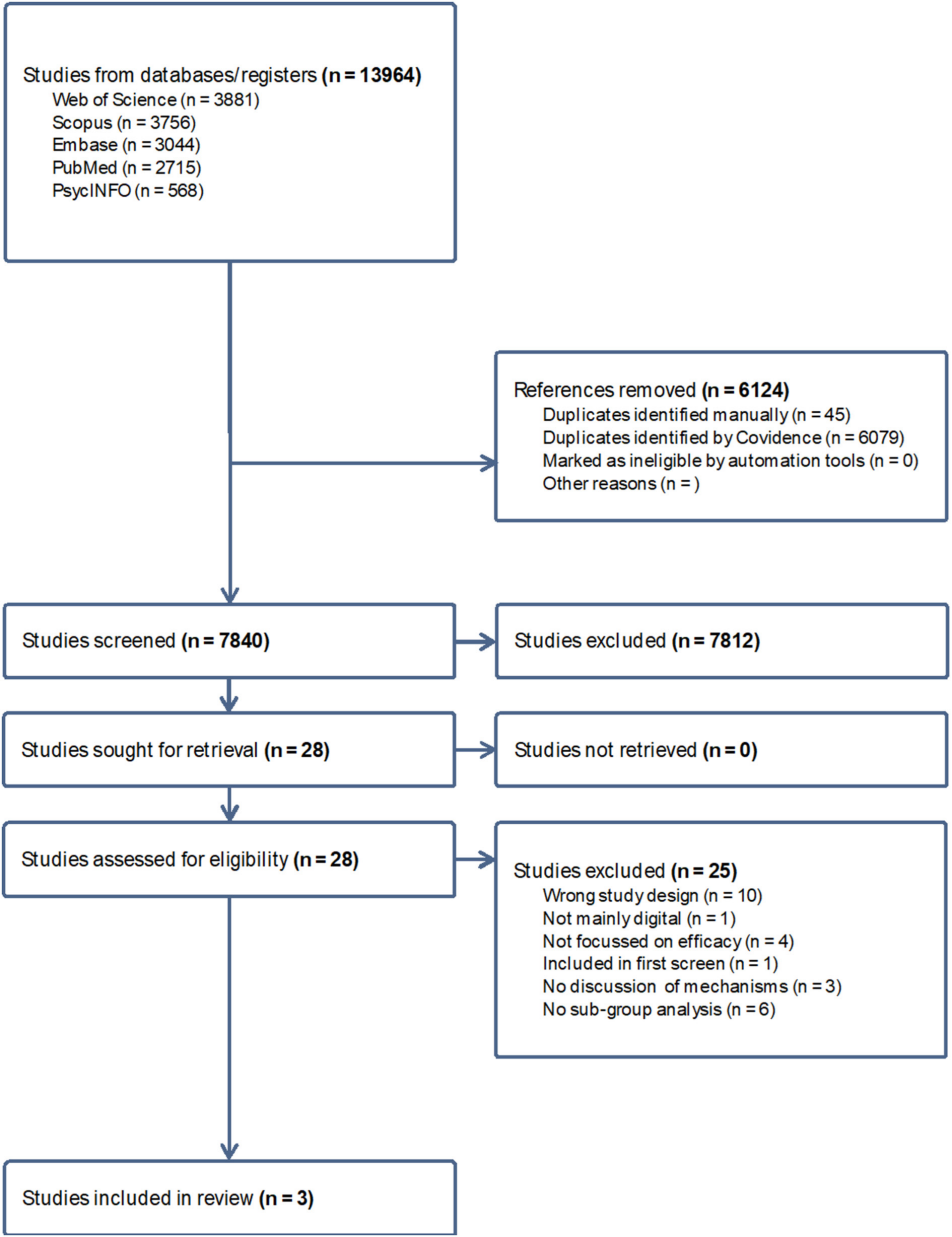
PRISMA‐ScR diagram (search conducted in June 2025).

The initial search generated 36,316 publications. After duplicates were removed, 17,503 were included in title and abstract screening. After screening, 104 articles were retrieved for full text review. Following full‐text screening, 83 further studies were excluded. The remaining 21 articles were included in the scoping review. The initial screening process is summarized in a PRISMA‐ScR study flow diagram (Figure 1). Results of the second search conducted in June 2025 are summarized in the PRISMA‐ScR study flow diagram in Figure 2. Details of the included studies from both searches can be found in Table 3.

**TABLE 3:** Summary of included studies.

Lead author (date), country, study design	Population	Inequality indicators	(1) Target behavior(s)*(2) Intervention components*	(1) Mechanisms discussed*(2) Mechanisms measured*	Findings
**Abdullah (2025)** [61], Japan, RCT	*N* = 174.30.5% female, 69.5% male. 58% were from low‐income households.	Income	PA (walking). *Activity tracking; visual feedback; financial incentives*.	Behavioral regulation; skills; environmental context and resources; motivation; knowledge.	Walking behaviors decreased from baseline in low‐income participants. Financial incentives insufficient to overcome structural barriers.
**Bennett (2014)** [62], United States, systematic review	Six trial studies met the inclusion criteria *N* = 4899.75% female, mean age 46.6 years. Included studies comprised > 50% ethnic minorities, or included subgroup analysis.	Racial/ethnic minority adults	Weight management. *Interactive digital interventions delivered* via *computer, web, text mobile phone or email or related technologies*.	Motivation.	eHealth interventions targeting weight‐loss in ethnic minority populations can result in small, short‐time effects. Engagement inconsistently measured. Scope for cultural tailoring of interventions is underexplored.
**Blackman‐Carr (2018)** [63], United States, RCT	*N* = 170. Overweight or obese African American and non‐Hispanic White women.	Ethnicity	Diet and PA. *Interactive Internet‐delivered components including lessons, video resources, self‐monitoring tools, exercise planning, and social message boards*.	Attitude toward the behavior; motivation; behavioral regulation. *Eating Behavior Inventory (EBI)*.	An Internet‐delivered weight loss intervention was less effective for African American women than non‐Hispanic White women. Log‐ins and changes in self‐regulation of eating scores partially mediated weight loss.
**Clark (2023)** [64], United States, systematic review	Nine studies met the inclusion criteria. The average mean age of the studies was 48.9 years, 80.4% were female, and 72.4% were ethnic minorities.	Income	PA and diet. *Telephone coaching, websites, interactive voice response, text messaging, social media/Facebook*.	Environmental context and resources; reinforcement.	Interventions tailored toward low‐income users were more effective. Financial incentives resulted in low‐income women losing three times more weight in one study.
**Cyriac (2021)** [65], United States, pilot study	*N* = 45. African American participants, 73% female, mean age 48.7 years.	Ethnicity	Diet and PA constructs. *A mobile lifestyle app, supplemented by Interactive Internet‐delivered lessons, goal setting, and self‐monitoring. Wearable fitness tracker to measure outcomes*.	Feedback processes; skills; behavioral regulation; knowledge; general attitudes and beliefs; environmental context and resources; social influences. *Diet self‐regulation (Health Beliefs Scale); social support for diet and exercise; perceived barriers to healthy diet*.	Culturally tailored diet and PA apps can improve diet and PA‐related psychosocial factors.
**Eisenhauer (2021)** [66], United States, RCT	*N* = 80. All male, mean age 54.2 years.	Geography (rural men)	Diet and PA. *A premium mobile lifestyle app, including self‐monitoring of diet and activity, goal setting, and personalized reports*.	Goals; needs; beliefs about capabilities; motivation; environmental context and resources; feedback processes; social influences. *Self‐monitoring of weight, diet, and activity; household size; education; income*.	Weight self‐monitoring was an effective means of improving weight loss in rural men.
**Flaherty (2020)** [67], Ireland, qualitative study	*N* = 10. All female, aged 30–45 years.	SES	Food purchasing. *A diet‐based lifestyle app including recipes, complemented by interactive Internet lessons*.	Self‐image; feedback processes; behavioral cueing; social influences; norms; beliefs about capabilities; goals; environmental context and resources; behavioral regulation; emotion; memory, attention, and decision processes.	mHealth apps can change food purchasing behaviors by disrupting existing habits and encouraging reflection, but self‐control also necessary.
**Frensham (2014)** [68], Australia, feasibility study	*N* = 8. Rural cancer survivors, aged 43–78 years. 75% female.	Geography (rural)	PA. *6‐week online walking program supported by a pedometer*.	Knowledge; motivation; emotion; feedback processes; goals.	Participants reported increase awareness of the health benefits of PA and motivation to walk
**Hornbuckle (2016)** [69], United States, pre‐post study	*N* = 46. Overweight and obese women on state support, aged 30–65 years.	SES	PA. *A 12‐week pedometer‐based walking intervention*.	Emotion; skills; beliefs about capabilities; social influences; memory, attention, and decision processes; reinforcement; environmental context and resources.	Pedometer‐based intervention increased average steps in low SES women at 3 and 12 months.
**Joseph (2016)** [70], United States, pre‐post study	*N* = 25 Overweight and obese young adult African American women, mean age 21.9 years.	Ethnicity	PA. *A culturally relevant Internet‐based application to monitor and promote physical activity*.	Behavioral regulation; social influences. *Screen time (Sedentary Behavior Scale); self‐efficacy for PA (Exercise Confidence Survey); Social Support for Exercise Scale; self‐regulation for PA (Health Beliefs Survey); Outcome Expectation Scale for Exercise; PA Enjoyment Scale*.	Internet‐enhanced PA intervention reduced screen time and enhanced SCT constructs, but did not increase PA.
**Kaur (2020)** [71], India, RCT	*N* = 668.73% female, mean age 52.7 years.	SES	Diet. *Text, email, social networking app, and smart eating website. Participants were also provided with a kitchen calendar and cooking utensils*.	Goals; values; social influences; environmental context and resources. *Education; occupation; housing; family size*.	SMART eating intervention improved diet behaviors in adults from diverse SES backgrounds.
**Kim (2020)** [72], South Korea, cross‐sectional study	*N* = 1718. South Korean adults, 50% female.	Education	Diet. *Various*.	Attitude toward the behavior; memory, attention, and decision processes; knowledge; motivation. *Education; household income; health status*.	Weaker association between heath app use and fruit and vegetable intake in higher SES populations.
**Marcus (2016)** [73], United States, RCT	*N* = 205. Inactive and overweight Latinas, aged 18–65 years	Ethnicity	PA. *Internet‐delivered, individually tailored PA intervention*.	Emotion; environmental context and resources; behavioral regulation; motivation; norms. *Adult literacy; Centre for Epidemiological Studies Depression Scale; Social Support for PA Scale; Physical Activity Enjoyment Scale; Neighborhood Environment Walkability Scale; Stage of Change; Self‐Efficacy for PA; Process of Change*.	Internet‐delivered, tailored intervention demonstrated greater improvements MVPA in Latinas than paper‐based equivalents.
**Myers‐Ingram (2023)** [74], United Kingdom, systematic review	Four experimental studies met the inclusion criteria. *N* = 373. Adults ≥ 18 years old with a BMI of > 25 kg/m [2].	SES	Weight management. *Various*.	Reinforcement; behavioral regulation.	Interventions effective in a small number of studies with small sample sizes. Unable to determine effectiveness of specific mechanisms due to heterogeneity.
**Pickett (2023)** [75], United States, cross‐sectional study	*N* = 6695.78% metropolitan. Gender not recorded.	Geography	PA. *A web‐based physical activity tracking platform. Personalized goal tracking, social sharing, and gamification*.	Social influences; environmental context and resources. *Metropolitan* vs *nonmetropolitan; group membership*.	While no differences were found across most indicators of PA, nonmetropolitan users showed less engagement with program, did fewer activities, and reported lower intensity exercise.
**Power (2019)** [76], United States, pre‐post study	*N* = 174. Post‐partum women. 83.9% Hispanic.	Ethnicity	Diet and PA. *A website including a web diary, links, weight management lessons, online message boards, instructional and inspirational videos, telenovela‐styled entertainment videos, and computer‐tailored feedback*.	Feedback process; environmental context and resources; motivation; social influences; needs; memory, attention, and decision processes; skills. *Self‐monitoring of weight, PA, diet*.	More frequent website visits and in‐person attendance predicted weight loss among completers at 12 months.
**Regnier (2018)** [77], France, qualitative study	*N* = 33.84.8% women.	SES	Diet. *A social network‐based cooking app for a low‐income population*.	Motivation; memory, attention, and decision processes; knowledge; environmental context or resources; norms; social influences; values. *Occupation; barriers to use; facilitators to use*.	Use of cooking apps in low SES groups has promise but is underexplored. However barriers remain high.
**Regnier** and **Chauvel (2018)** [78], France, qualitative study	*N* = 79.76% women, aged between 23 and 70 years.	SES	Diet and PA. *Self‐tracking fitness smartphone apps*.	Motivation; feedback processes; skills; values; norms; perceived susceptibility or vulnerability; social influences; needs; environmental context and resources. *Occupation*.	Users in lower SES groups less likely to use health apps and engage in self‐quantification.
**Spelt (2019)** [79], Europe, RCT	*N* = 95. Adults from Greece and the Netherlands. 68% female.	SES	PA. *Smartphone app and a wearable activity tracker*.	Behavioral cueing; skills; knowledge; beliefs about capabilities; behavioral regulation; motivation; environmental context or resources. *Education; household composition; well‐being; attitudes; intention; perceived behavioral control*.	6‐week lifestyle eCoaching app successful increased PA in low SES populations at 19‐weeks. No differences observed along cultural (Greece vs. Netherlands) or SES lines.
**Szinay (2023)** [40], Europe, systematic review	Studies ranged from 48 to 251,718 participants. Adults with no pre‐existing medical conditions.	SES, ethnicity, occupation, income, education, geography, age, gender.	Weight‐related behaviors. *Mobile applications. No face‐to‐face components on nonmobile digital technology*.	Norms; environmental context and resources; skills; knowledge.	Limited/mixed evidence of a digital divide in exclusively mobile interventions targeting weight‐related behaviors. Reporting results by inequality indicators is inconsistent.
**Toon (2022)** [80], United Kingdom, pre‐post study	*N* = 27,248.5.3% male, mean age 41, mean BMI of 33.	Socio‐economic	Diet. *A national Internet‐based weight loss program*, *including goal setting and self‐monitoring*.	Optimism; goals; memory, attention and decision processes; motivation. *Engagement*.	An national online program can support weight‐loss across SES backgrounds. Increased engagement leads to greater weight‐loss.
**Volz (2021)** [81], United States, pre‐post study	*N* = 260. Adults in the United States, 79% female, 60% non‐Hispanic White	Income	Diet and PA. *Initial face‐to‐face study, website lessons, and self‐monitoring using a fitness app*.	Emotion. *Education; income; emotional regulation (stress)*.	Income positively associated with weight loss, mediated through stress, in an online weight loss program.
**Wayne (2015)** [82], Canada, RCT	*N* = 131. Adults with type 1 diabetes, 70% women, 66% with a household income of less than CAN $50,000	SES	Diet and PA. *Health coaching + smartphone, including tracking*.	Emotion; reinforcement. *Life satisfaction; anxiety; depression; positive/negative affect; education; employment; income; car access*.	mHealth accelerated improvements in glucoregulation in low‐SES when combined with in‐person health coaching. No differences seen between mHealth supplemented and coaching‐only groups at 6 months.
**Western (2021)** [39], United Kingdom, systematic review	*N* = 5419	SES	PA. *Any web‐based interface or wearable device that communicates information to the user, any mobile‐based program, or offline computer program*.	Environmental context and resources; behavioral regulation.	There is no evidence that digital PA interventions are effective in low SES populations.

### 3.1 Study Type

Of the included studies, seven are randomized controlled trials (RCTs) [61, 63, 66, 71, 73, 79, 82], five are *pre‐pos*t studies [69, 70, 76, 80, 81], five systematic reviews [39, 40, 62, 64, 74], three are qualitative [67, 77, 78], with the remainder cross‐sectional [72, 75], pilot [65], or feasibility studies [68]. RCTs either compared a DBCI with a no intervention control group [79], compared nondigital (or enhanced digital) intervention [61, 66, 73, 82], or explored differences in their effectiveness between demographic groups [63, 71] with follow‐up periods from 4 [63, 79] to 6 months [71, 73, 82]. One study had no postintervention follow‐up [61]. *Pre‐post* designs measured changes in behavior [69, 76, 80] or psychometric constructs [70, 81] before and after an intervention with two studies also including follow‐up at 12 months [69, 76]. Systematic reviews sought to assess the effectiveness of DBCIs across different disadvantaged groups. Qualitative studies either focused on low‐SES populations [67, 77] or reported differences by socioeconomic background [78].

### 3.2 Populations

The majority of studies were conducted in high‐income countries including the United States (*n* = 11) [62, 63, 64, 65, 66, 69, 70, 73, 75, 76, 81], Europe (*n* = 8) [39, 40, 67, 74, 77, 78, 79, 80], Australia (*n* = 1) [68], South Korea (*n* = 1) [72], Canada (*n* = 1) [82], and Japan (*n* = 1) [61]. One study was conducted in India [71]. Most studies had a strong female bias, with a majority of studies either focused exclusively on female populations [63, 67, 69, 70, 73] or including samples consisting of at least two thirds female participants [62, 64, 68, 71, 74, 75, 76, 77, 78, 79, 80, 81, 82]. One RCT reported a sample consisting of 80% male participants [61]. Included studies focused on those of low SES or one of its components (*n* = 15) [39, 40, 61, 64, 67, 69, 71, 72, 74, 77, 78, 79, 80, 81, 82], ethnic minorities (*n* = 6) [62, 63, 65, 70, 73, 76], or those living in rural communities (*n* = 3) [66, 68, 75]. Three studies focused on populations with pre‐existing health conditions [68, 82].

### 3.3 Intervention Design

Most interventions were delivered through the Internet or smartphone applications, with studies involving wearables featuring in just four studies [65, 68, 69, 79]. Typically, both Internet and mobile delivered interventions included facilities for goal setting, self‐tracking, education, and sharing information. The weight‐related behaviors most commonly targeted were increasing PA [39, 61, 68, 69, 70, 73, 75, 79], improving diet [67, 71, 72, 80], or a combination of both [40, 62, 63, 64, 65, 66, 74, 76, 77, 78, 81].

### 3.4 Psychosocial Mechanisms

**FIGURE 3:**
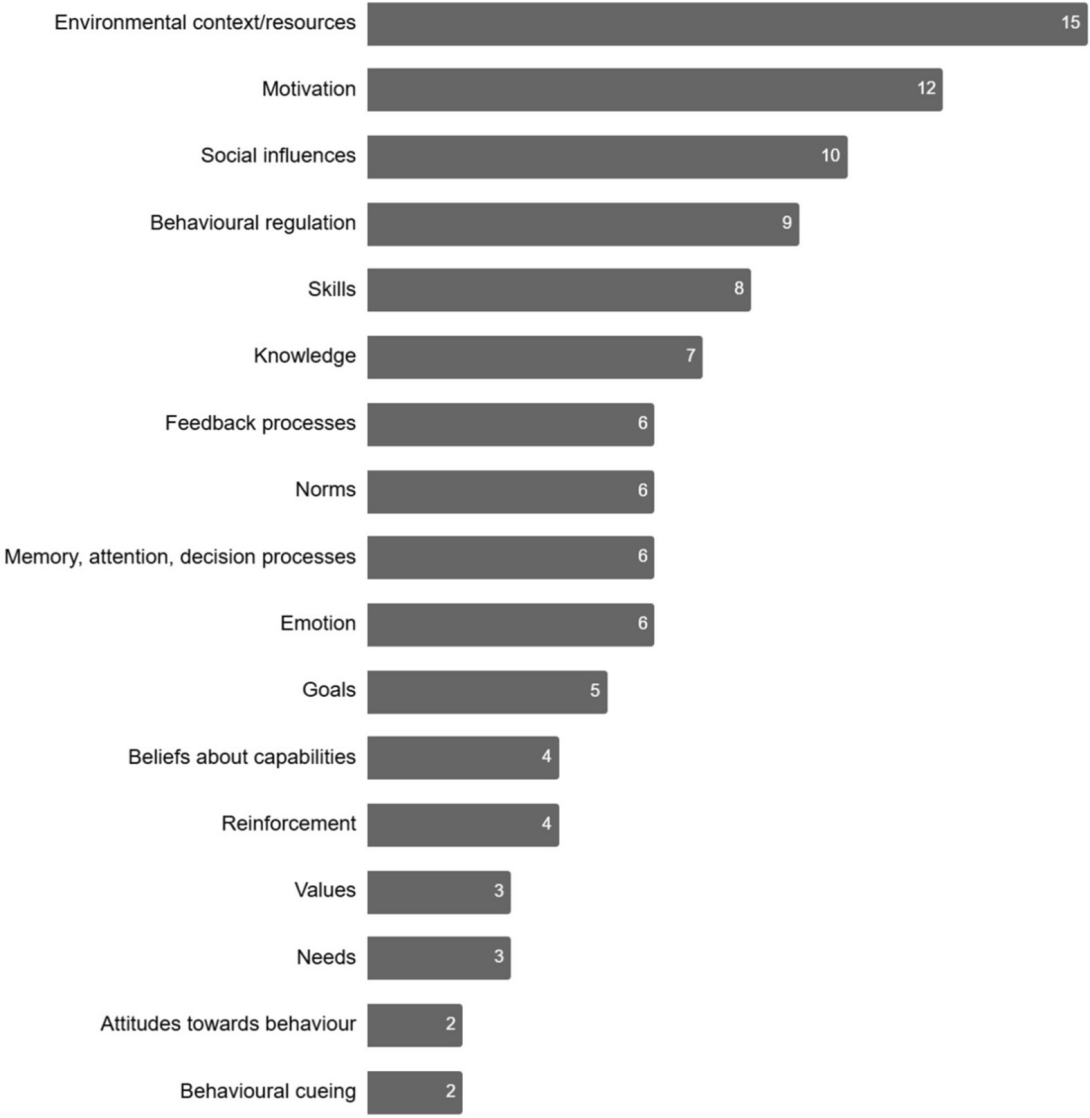
Mechanisms of action (MOAs) discussed in two or more papers.

Seven of the 21 studies sought to measure potential psychosocial mechanisms empirically [63, 65, 70, 73, 79, 81, 82]; however, formal moderation or mediation analyses were conducted in only two studies [63, 81]. The remainder explored correlations between changes in psychometric scores and outcomes. The most common mechanisms were related to *environmental context and resources* (*n* = 15), *motivation* (*n* = 12), and *social influences* (*n* = 10). Figure 3 summarizes the mechanisms that were either measured or discussed in the included papers.

### 3.5 Environmental Context or Resources

Fifteen of the 24 studies discussed environmental context and resources as mechanisms relevant to the digital health divide [39, 40, 61, 64, 65, 66, 67, 69, 71, 73, 75, 76, 77, 78, 79]. These included perceived environmental barriers to offline PA behavior such as fears about crime, traffic, or the availability of pavements [69] and the restricted availability of fruit and vegetables, which impacted peoples' ability to act upon the DBCI guidance [71]. Differences in digital infrastructure were also highlighted where the slowness or capacity issues of devices had a significant impact on engagement and satisfaction of low‐income users of DBCIs for health [77, 78]. While access to technology is often understood as binary, nuances in access were identified as important, such as when access to devices may vary spatially (e.g., work vs. home) or temporally such as when technologies are available during the day but not in the evening [66]. Mixed evidence emerged as to how technology use varied between disadvantaged groups. Thus, while one study found that African American women were more likely to be minimal versus consistent users of the Internet [63], another found engagement rates in ethnic minority adults consistent with the wider intervention evidence base [62].

### 3.6 Motivation and Emotion

The role of motivation in the effectiveness of DBCIs was discussed in 12 studies [61, 62, 63, 66, 68, 72, 73, 76, 77, 78, 79, 80]. One way in which motivation can manifest is through engagement [80]. For example, one Internet‐based intervention found that fewer logins from African American women were found to mediate the racial disparity in weight loss outcomes [62]. Furthermore, noncomputer owners given a computer or Internet access as part of an intervention remained less likely to log on than those who already owned a computer [76]. Six studies explored the relationship between individual affect and emotion and the use and effectiveness of DBCIs [67, 68, 69, 73, 81, 82]. For example, one study found that income‐based stress inhibited the efficacy of a DBCIs, suggesting that employing a stress intervention first might improve the efficacy of subsequent weight‐related DBCIs [81]. Furthermore, the low‐SES women interviewed in a study exploring the efficacy of a 12‐month pedometer‐based intervention reported that existing emotional and mental health challenges impeded their engagement with digital health [69]. Researchers also explored whether DBCIs might offer scope to navigate the influence of affect on engagement in weight‐related behaviors. For example, one qualitative study found that a health app could redirect emotion‐driven purchasing behaviors toward higher order goals of health and well‐being [67], and a three‐tiered goal system, which was responsive to affective state, was well received in a pedometer‐based walking intervention with rural cancer survivors [68].

### 3.7 Social Influences and Norms

Three studies directly measured subjective social support [65, 70, 73]. Of those, one study targeting an African American population found that increased social support for diet and PA had moderate positive correlations with fruit and vegetable consumption and moderate PA per week. However, the remaining two studies targeting African American and Latina women found no significant changes in social support during the intervention [70, 73]. While there was limited qualitative support for the use of online forums and Facebook groups [65, 66], the rural male participants in one study disliked the asynchronous nature of online discussion boards, suggesting that more timely, personalized interactions would be preferred [66]. However, a lack of social support was argued to have impacted on participants' ability to reach step goals in a 12‐month pedometer‐based intervention aimed at women of low SES [69]. Household composition may also help mitigate disparities in the use and efficacy of DBCIs, with children playing a facilitative role in supporting parents' use of health apps [39]. A desire to maintain family harmony and resolve conflicting food goals among family members was argued to constrain healthier purchasing behaviors promoted by an app [74].

### 3.8 Skills, Memory and Attention Processes

The effective use of DBCIs depends on users possessing some existing skills. For example, one study argued that obese users may find performing the activities required by DBCIs for PA harder or more discomforting, suggesting that weight loss be targeted first through a diet intervention [69]. Targeting cooking skills was successful at reducing fat, sugar, and salt intake and improving fruit and vegetable consumption across the SES spectrum in an intervention, which paired a recipe website, electronic messaging, and social media with cooking utensils in a North Indian city [71]. However, a qualitative study in a low‐income population in France found that existing cooking skills could also be a barrier, with those with the most interest in cooking showing the least interest in a digital app [77]. In the two qualitative studies by Regnier and colleagues, digital skills were cited as a barrier to engagement with a cooking app in [77, 78]. Attentional capacity to use and engage with digital health was discussed in several papers suggesting shorter, more frequent engagements may be more effective [69]. The ability to attend to digital health versus other competing priorities was seen as a particular challenge for low SES participants [67]. However, in a cross‐sectional study conducted in South Korea, the use of more than one health app was positively associated with increased fruit and vegetable consumption, suggesting multiple interventions may be preferable to using a single app [72].

### 3.9 Feedback Process, Behavioral Regulation, Goals, and Reinforcement

Tracking and feedback were common components of DBCIs for health being evaluated. For example, one intervention targeted nutrition tracking in their mHealth intervention to improve weight‐related behaviors in an African American population [65]. However, while the use of digital scales was associated with weight loss in a study of rural men [66], several studies found no correlation between weight tracking and weight loss [68, 70]. Furthermore, in one qualitative study, low SES users reported being less comfortable with engaging with self‐tracking than those in higher social milieus [78]. Five studies explored the relationship between constructs related to self‐regulation of behaviors and the effectiveness of DBCIs in disadvantaged groups [63, 65, 70, 73, 79]. For example, changes in the *Eating Behavior Inventory* partially mediated weight loss in a study comparing African American women with non‐Hispanic White women [63]. However, the evidence is mixed. For example, one study found improvements in PA, but not in PA‐related constructs including intention, attitude, or perceived behavioral control [79], whereas another found that DBCIs could successfully target regulatory constructs, but this did not result in improved weight‐related outcomes [65]. Evidence on the use of financial incentives to reinforce behaviors was mixed. While one systematic review included a study where women offered financial incentives achieved three times the weight loss of those not offered an incentive [64], participants in a 12‐week pedometer‐based walking intervention who were offered a cash reward did not increase their daily step counts [69]. Similarly, financial incentives were insufficient to overcome structural barriers in an intervention designed to increase walking behaviors [61].

## 4 Discussion

The 24 studies included in this scoping review highlight how researchers have explored the mechanisms which might explain the divide in the effectiveness of DBCIs for weight‐related behaviors. The majority of the papers (18/24) were published in the United States or Europe, likely due to an increased focus on digital health technologies in developed countries [83]. While the social and digital determinants of health inequality are well described in the wider literature [3, 84], the small number of papers, which met the inclusion criteria suggest that too few studies have investigated the causal mechanisms through which determinants are translated into outcomes. This is in keeping with reviews, which have found the field to be dominated by descriptive rather than explanatory research [37, 85]. Furthermore, most of the papers were quantitative, with just three qualitative studies meeting the inclusion criteria. This is a concern as given the complex systems surrounding digital health use, qualitative research is well positioned to elucidate the processes through which the socio‐, economic, and digital determinants of health manifest into disparities of outcomes [84].

The review found that mechanisms related to environmental and social contexts, motivation and social influences were most frequently cited. In terms of social contexts, one proposed solution to improving engagement in digital health in ethnic minority populations has been to culturally tailor interventions [86]. However, the studies included here present only limited support for such approaches. For example, an app co‐designed with members of an African American church congregation was found to be effective at promoting weight loss [65], whereas the telenovela included in an intervention aimed at—but not developed with—a Latina community was largely ignored [73]. This is consistent with the wider evidence base, with one 2021 systematic review of 23 papers finding that cultural tailoring offered some promise [87], while another found either small effect sizes or nonsignificant results leading the authors to suggest that cultural tailoring was “not worth the effort” [88]. Here, it is possible that it is the co‐design of interventions with target populations, rather than cultural tailoring per se, which is important; however, evidence assessing the effectiveness of co‐design in digital health is lacking.

In terms of motivation, lower socioeconomic participants report that a range of competing priorities—financial concerns, lack of transport, family issues, and mental health—have an impact on their motivation to engage with digital health [69, 77, 78]. These reflect some of the structural inequalities that have been found to drive digital exclusion in other reviews [89]. Negative affect and emotion are also likely to be important mechanisms influencing engagement in weight‐related behaviors [90]. However, there was only limited exploration of the interaction between SES and affect in the included studies, which represents a gap. For example, those interventions targeting improvements in well‐being reported mixed results, with some studies noting improvements while using a health app [79], while others found no improvement in self‐reported stress or depression [73]. Furthermore, given the absence of formal mediation or moderation analyses, it is not clear whether improvements in these psychosocial constructs led to improved weight‐related behaviors. That said, one study found that stress fully mediated the relationship between income and engagement in a digital health app and partially mediated the relationship between income and weight loss [81]. One solution to this might be to adopt tiered goal systems that are responsive to daily changes in affect as suggested in Frensham et al. [68].

Another finding of interest was the relationship between SES and attitudes toward self‐tracking. Feedback and self‐monitoring are theorized to support self‐efficacy by providing information on individual performance and progress, thereby increasing task motivation [91]. However, the qualitative studies included here suggest that individuals from lower social milieus might be both less comfortable and less adept at using digital self‐tracking technologies [78]. Despite this, there is only limited exploration in the literature of the sociocultural perspectives of digital self‐quantification [92]. Indeed, while there is increased awareness of the ways in which digital tracking technologies can be seen as both empowering and disempowering, and about our sometimes ambivalent and contradictory use of digital technologies [93], in the context of DBCIs for health, more research is required to understand the socioeconomic dimensions of digital self‐tracking. Furthermore, research indicates that not all potential users appreciate self‐tracking as a means of changing behavior independent of social inequality indicators, so alternative routes might need to be explored to increase uptake and engagement with these technologies generally [94, 95].

However, the included papers reflect also a lack of standardization in how MoA are conceptualized and described, resulting in findings that were often preliminary, inconclusive, or contradictory. For example, despite the availability of validated scales to measure social support for health behaviors, only three of the nine studies citing social influences sought to measure them empirically [65, 70, 73]. This is consistent with the wider behavioral change evidence base, which often uses inconsistent and heterogeneous measures, impeding progress in understanding what makes interventions effective [96]. This study adds further weight to the need to adopt a standard framework of concepts and terms, such as those set out in the MoA and related behavioral change ontologies [57]. Furthermore, while a limited number of studies explored the efficacy of DBCIs for those in two or more disadvantaged groups, too few studies examine how multiple disadvantages compound and intersect to give rise to new, or amplify existing, health inequalities [97, 98]. Given those limitations, it is not possible to draw firm conclusions about what mechanisms are likely to be most effective in DBCIs, or indeed explain the lack of an effect, across different domains of disadvantage.

The main strengths of this review are the comprehensive and systematic backward citation screening of literature from multiple databases, using multiple researchers, and the use of contemporary frameworks and taxonomies to categorize and interpret findings. Importantly, the review also has a few limitations. First, it is exploratory and the limited number of studies, which measure mechanisms, and inconsistencies in how they are conceptualized, prevent empirical analysis of effect sizes in the form of a systematic review or meta‐analysis. As such, the relative effectiveness of specific mechanisms across different cultural or geographic contexts could not be established, or indeed between digital and nondigital interventions. It is also unlikely to be a comprehensive survey on all the possible mechanisms that account for the digital divide, given the small numbers of papers that met the inclusion criteria. Further systematic research, not only into whether a digital health divide exists across a range of social inequality indicators [99] but also into its underlying mechanisms in various populations is thus urgently needed. Furthermore, while effort was taken to map the results to the MoA framework, deductive coding of content relied on authors' descriptions of mechanisms, which did not always map exactly to the framework's vocabulary. Finally, the limited number of qualitative studies, which met the inclusion criteria constrained our ability to explore the pathways through which social and digital determinants lead to disparities of outcomes across different disadvantaged groups.

## 5 Conclusion

In conclusion, this scoping review identified the nascent body of research into the mechanisms that underpin the digital divide in the effectiveness of DBCIs for weight‐related behaviors. The key takeaway from this review is that MoA are inconsistently conceptualized and measured, making it difficult to identify what intervention components are most effective in targeting disadvantaged groups. The included studies highlight how researchers have employed mechanisms such as cultural tailoring, social support, and improving affect to tackle the digital divide. Those from lower socioeconomic backgrounds may be less comfortable and less adept at using the self‐tracking requirements of digital health. However, too often the evidence is either primary, inconsistent, or contradictory. In addition to adopting standardized definitions of mechanisms, systematic reviews should be undertaken to explicitly compare the effectiveness of different MoA in both digital and nondigital contexts, and investigate underrepresented MoA.

## Author Contributions

L.C.M. wrote the scoping review protocol, performed the electronic database search, undertook title and abstract screening for all articles, performed the full article review, extracted data from the included studies, wrote the first draft of the manuscript, and undertook subsequent revisions; M.A.‐M. and V.R. were second reviewers on all papers screened. M.A.‐M. was the sole second reviewer during full article screening. M.A.‐M. quality checked data extraction. L.M.K. and M.W. conceptualized the review and provided feedback on the Scoping Review Protocol, and provided feedback on all drafts of the manuscript. M.W. was the primary supervisor of L.C.M. L.C.M. and M.W. had full access to the data and accepted responsibility for its accuracy. L.C.M., L.M.K., and M.W. screened all papers in the 2025 update search. Conflicts were discussed by L.C.M., L.M.K., and M.W. and resolved. L.C.M. and M.W. conducted a full‐text review. L.C.M. coded the papers identified in the supplementary search conducted in June 2025, which was subject to quality checking by M.W. L.C.M. redrafted the manuscript based on the updates.

## Funding

L.C.M.'s PhD is funded by the Economic and Social Research Council (Ref. ES/P000630/1). The funders had no role in the study design, data collection, and analysis, decision to publish, or preparation of the manuscript. All authors had full access to all of the study data, and the corresponding authors had the final responsibility for the decision to submit for publication.

## Conflicts of Interest

The authors declare no conflicts of interest.

## Supporting information


**Data S1:** Supporting Information.

**Data S2:** Supporting Information.

## Data Availability

Data sharing not applicable to this article as no datasets were generated or analysed during the current study.
