# Supplementary material for: A Scoping Review of the Mechanisms Influencing Socioeconomic Disparities in Outcomes of Digital Interventions for Weight‐Related Behaviors
Source: Obes Rev. 2026 Mar 12;27(8):e70121. doi: 10.1111/obr.70121 (PMC13371839; doi:10.1111/obr.70121)
Supplement: Supplementary file 1 — Data S1: Supporting Information. [file OBR-27-e70121-s002.pdf]

Search Strategy

Key concept	**Embase** (Title or Abstract)	**PubMed** (Title/Abstract)	**APA PsychInfo** (Abstract)	**Web of Science** (Abstract)	**Scopus** (TITLE-ABS-KEY)
1. Health	health OR "physical activity" OR diet OR fitness OR weight OR exercise OR nutrition OR "body-weight" OR obese	health OR "physical activity" OR diet OR fitness OR weight OR exercise OR nutrition OR "body-weight" OR obese	health OR "physical activity" OR diet OR fitness OR weight OR exercise OR nutrition OR "body-weight" OR obese	health OR "physical activity" OR diet OR fitness OR weight OR exercise OR nutrition OR "body-weight" OR obese	health OR "physical activity" OR diet OR fitness OR weight OR exercise OR nutrition OR "body-weight" OR obese
2. Inequalities	inequalit* OR disparit* OR disadvantaged OR depriv* OR socioeconomic OR "socio economic" OR ses OR poverty OR "digital divide"	inequalit* OR disparit* OR disadvantaged OR depriv* OR socioeconomic OR "socio economic" OR ses OR poverty OR "digital divide"	inequalit* OR disparit* OR disadvantaged OR depriv* OR socioeconomic OR "socio economic" OR ses OR poverty OR "digital divide"	inequalit* OR disparit* OR disadvantaged OR depriv* OR socioeconomic OR "socio economic" OR ses OR poverty OR "digital divide"	inequalit* OR disparit* OR disadvantaged OR depriv* OR socioeconomic OR "socio economic" OR ses OR poverty OR "digital divide"
3. Digital	"digital devices" OR "mobile phone" OR cellphone OR "mobile app" OR smartphone OR "smart phone" OR app OR eHealth OR "e-health" OR mHealth OR "m-Health" OR "fitness tracker*" OR pedometer* OR "step-track*" OR "text message*" OR "web-based" OR website OR internet OR online OR telemedicine OR "tele-medicine"	"digital devices" OR "mobile phone" OR cellphone OR "mobile app" OR smartphone OR "smart phone" OR app OR eHealth OR "e-health" OR mHealth OR "m-Health" OR "fitness tracker*" OR pedometer* OR "step-track*" OR "text message*" OR "web-based" OR website OR internet OR online OR telemedicine OR "tele-medicine"	"digital devices" OR "mobile phone" OR cellphone OR "mobile app" OR smartphone OR "smart phone" OR app OR eHealth OR "e-health" OR mHealth OR "m-Health" OR "fitness tracker*" OR pedometer* OR "step-track*" OR "text message*" OR "web-based" OR website OR internet OR online OR telemedicine OR "tele-medicine"	"digital devices" OR "mobile phone" OR cellphone OR "mobile app" OR smartphone OR "smart phone" OR app OR eHealth OR "e-health" OR mHealth OR "m-Health" OR "fitness tracker*" OR pedometer* OR "step-track*" OR "text message*" OR "web-based" OR website OR internet OR online OR telemedicine OR "tele-medicine"	"digital devices" OR "mobile phone" OR cellphone OR "mobile app" OR smartphone OR "smart phone" OR app OR eHealth OR "e-health" OR mHealth OR "m-Health" OR "fitness tracker*" OR pedometer* OR "step-track*" OR "text message*" OR "web-based" OR website OR internet OR online OR telemedicine OR "tele-medicine"
4. Efficacy/ engagement	effectiv* OR engag* OR use OR usage OR effic* OR adoption	effectiv* OR engag* OR use OR usage OR effic* OR adoption	effectiv* OR engag* OR use OR usage OR effic* OR adoption	effectiv* OR engag* OR use OR usage OR effic* OR adoption	effectiv* OR engag* OR use OR usage OR effic* OR adoption
